# Oil Palm Germplasm Resources and Their Conservation: Advances and Challenges for In Vitro Conservation

**DOI:** 10.3390/plants14233631

**Published:** 2025-11-28

**Authors:** Saeed Rauf, Rodomiro Ortiz, Yong Wang

**Affiliations:** 1 State Key Laboratory of Tropical Crop Breeding/Coconut Research Institute, Chinese Academy of Tropical Agricultural Sciences, Wenchang 571339, China; saeedbreeder@hotmail.com; 2 Department of Plant Breeding & Genetics, College of Agriculture, University of Sargodha, Sargodha 40100, Pakistan; 3 Department of Plant Breeding & Genetics, Swedish University of Agricultural Sciences, 23422 Alnarp, Sweden; rodomiro.ortiz@slu.se

**Keywords:** DMSO, genetic stability, propagule, somatic embryoids, vitrification

## Abstract

In vitro germplasm conservation provides an alternative method for preserving plant species that are vulnerable to natural hazards or for which in situ conservation is costly and challenging to manage. This review examines the significance and challenges associated with various in vitro conservation methods. It also provides an overview of the current advances in cryopreservation technology for oil palm. In vitro conservation approaches include two strategies: medium-term conservation, in which plants are maintained through the slow growth of explants, facilitated by the gradual release of nutrients, and low-temperature storage. The second approach involves long-term preservation via cryopreservation in liquid nitrogen. Cryopreservation enables the storage of pollen, calli, somatic embryos, and zygotic embryos. Significant progress has been made in cryopreservation, which was initially limited to cold-tolerant species. New techniques focus on conserving sensitive species, such as oil palm, through rapid dehydration and vitrification procedures using various plant materials, particularly polyembryoids and zygotic embryos. Additionally, hardening of plant material is to be induced through pre-culture techniques to enhance their survival under osmotic stress and ultralow temperature. The mechanisms underlying the adaptability of various plant materials, i.e., somatic embryoids and zygotic embryos under cryopreservation, need to be understood.

## 1. Introduction

Oil palm (*Elaeis guineensis* Jacq.) plays a vital role worldwide, providing an essential source of cooking oil and vegetable protein for humans and animals. The edible oil is extracted from the fruit mesocarp, which has a high oil content (upto 50%) in elite hybrid cultivars. This oil is excellent for deep frying due to its high saturated fatty acids, particularly palmitic and stearic acids [1]. Palm kernel oil is of lower quality and is mainly used in soap production and as a cosmetic ingredient. Compared to other tropical industrial crops, oil palm offers greater profits to farmers, making it more desirable. Two species make up the genus or class Elaeis: *Elaeis guineensis* Jacq. and *Elaeis oleifera* (Kunth) Cortés, both of which can cross through natural or artificial hybridization. *E. guineensis* is from Western and Central Africa, while *E. oleifera* is from South America [1]. There are numerous problems related to the research and production of oil palm. It is vulnerable to insect infestation, pathogen attack, and drought stress, making them susceptible to losses during the early phases of development [2]. Moreover, there is a need to enhance adaptability to diverse climates and expand the genetic potential of the species. This can be done by harnessing the functional diversity within germplasm resources to address production constraints in oil palm cultivation.

Genetic resources can be used in breeding programs once the target germplasm accessions are identified through characterization. This program is integral to the strategic goals of germplasm collection, conservation, and characterization. The safeguarding of germplasm relies on robust conservation efforts, including the protection of natural habitats, establishment of field banks, and conservation of germplasm under controlled conditions, which range from seed banks to in vitro conservation methods.

Against this background, this review emphasizes the importance of germplasm conservation and its pivotal role in improving oil palm species, and highlights advancements in in vitro germplasm conservation. Additionally, this review provides insights into the challenges and the development of new genotype-independent protocols.

## 2. Why Is There a Need to Conserve Oil Palm Germplasm

**Figure 1:**
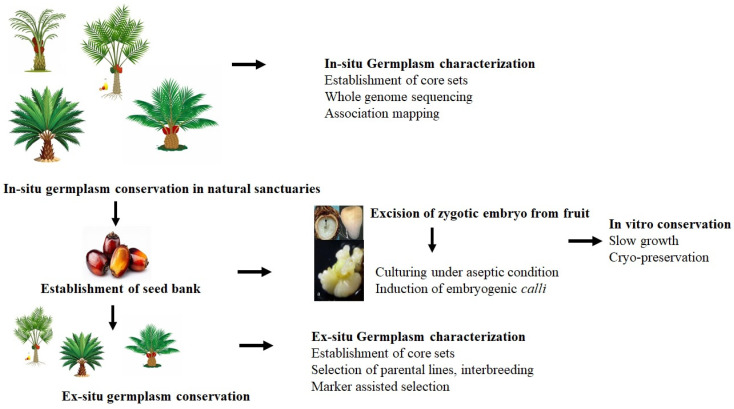
Integrated approaches for germplasm conservation, characterization, and utilization.

Conserving oil palm germplasm resources is crucial for preserving genetic diversity and ensuring the crop’s long-term sustainability in the current global climate change scenario. Traditionally, germplasm has been conserved in natural sanctuaries or field banks (Figure 1). Genetic diversity within these conserved sites is assessed using various morphological and molecular markers to quantify genetic variation. This information helps formulate future strategies for conserving, utilizing, and managing oil palm germplasm resources.

Diversity within oil palm germplasm has been assessed using isozymes, which revealed high genetic differentiation (FST = 0.301) among West African germplasm [3]. The genetic diversity of oil palm populations conserved in “ex-situ” collections diminishes progressively from Costa Rica towards Honduras and southeast Colombia [4].

Molecular markers were also used to determine population structure; specifically, genetic differentiation was estimated using established sequence tags (ESTs) with a value of 0.25 [4]. A genetic distance of 0.77 was observed in a study involving a germplasm collection from 10 African countries in 3 breeding populations and 1 semi-wild population, which were assessed through SSR analysis [1,5]. This study identified the genotypes from Madagascar as genetically distinct from those in other regions, such as Ghana and Côte d’Ivoire [5]. The populations of Madagascar were distinct, indicating potential diversity and independent evolutionary histories. Genetic diversity within Nigerian germplasm was assessed using simple sequence repeat (SSR) markers. Genetic differentiation among germplasm populations was moderate (Fixation term, FST = 0.12), and a core germplasm subset of 58 oil palm accessions was identified, representing 31% of the genetic diversity in Nigerian germplasm [6].

Human activities and environmental factors significantly affect these African wild populations, and ongoing destruction from biological or ecological factors could reduce their genetic diversity. Therefore, there is a dire need for germplasm conservation for future exploitation in breeding programs.

## 3. Utility of Oil Palm Germplasm Resources in Breeding Programs

Oil Palm breeding is a multifaceted process based on several ambitious breeding objectives, including heterosis, disease resistance, modification of fatty acid profiles, environmental stress tolerance, and adaptability. However, achieving these objectives is unlikely without adequate genetic variability. Conventionally, oil palm is improved through reciprocal recurrent selection, in which individual plants are selected within each group, such as Dura (thick, fibrous mesocarp with a hard shell surrounding the oil-rich kernel, lower oil extraction rates from the fruit pulp but higher fruit yield components) and Pisifera (large mesocarp and a very thin, hard shell, higher oil yield from the mesocarp (fruit pulp), small kernel and low fruit yield components). Selected individuals are also evaluated for their ability to combine through progeny testing. Superior plants are crossed to develop “tenera” hybrids with better oil content. A selection gain of 18% per cycle has been observed in reciprocal recurrent selection. Germplasm resources have been evaluated based on morphological traits, such as fruit shell forms (thin, thick), exocarp color, and plant characteristics, i.e., plant height and fruit bunches related to oil palm improvement [1]. Traits such as rachis length and nut mass exhibit high heritability (60%) and can be effectively utilized as selection criteria, as they also display a high coefficient of variability (0.72) [7]. MOPB-Angula germplasm exhibits high genetic variability in terms of bunch yield and oil quality components [8]. There is considerable variability in these traits, and “Dura” progenies, such as AGO 02.02, exhibited the highest fresh fruit bunches (240 kg tree^−1^) and oil yield (29.46 kg tree^−1^) [8]. The genetic gain for fresh fruit bunches and the number of bunches per plant was 19% [9].

Elite germplasm resources enable rapid improvement of the species by expanding yield potential and other desirable traits, such as early maturity, increased fruit-bearing branches, a higher proportion of mesocarp in the fruits, the number of fruits per bunch, and higher oil content. However, breeding goals such as host plant resistance to pathogens and insects, as well as growth modifications, require the exploitation of secondary and wild germplasm of oil palm. *E. oleifera* is used as a parental species for improving host plant resistance to pathogens; however, this species is not cultivated on a large scale due to its poor yield potential (i.e., less than 1 t ha ^−1^, compared to 3–4 t ha^−1^ for *E. guineensis*) [10]. The widespread cultivation of a single genotype has led to disease epidemics, necessitating the exploitation of novel genetic resources to improve the oil palm [2].

Between 1970 and 2000, oil palm breeding benefited from the use of genetic resources, resulting in disease-resistant germplasm, particularly against *Fusarium* wilt, and dwarf genotypes. During this period, utilization of genetic diversity within oil palm germplasm increased by 42% across various breeding programs in Africa and South America [11].

African genetic resources have contributed to the development of dwarf types (from Nigeria), large dura fruit [12], and improvements in iodine value and oleic acid content during the second and third breeding cycles [6,13]. Germplasm from Tanzania has contributed to favorable traits, including a high harvest index, thin-shelled tenera, and high fruit bunch yield [12,14,15].

The ongoing exploitation and use of genetic resources will further support the breeding of new ideotypes with improved resistance to stress factors and enhanced adaptability to evolving farming systems. However, to fully realize the benefits of these germplasm resources, it is essential to implement effective conservation strategies across diverse systems, ensuring their long-term sustainability and enabling their continued use in future breeding programs.

## 4. Integration of Ex Situ Germplasm Resources with In Vitro Conservation Strategies

In situ germplasm conservation faces several challenges, including spatial constraints on growth, urban expansion, land deterioration, climate change (extreme temperatures and unpredictable rainfall), and the onset of diseases and pests at collection sites [6]. Ex situ conservation also has problems, including the need to purchase land and other resources, as well as the costs of agronomic management and farming practices, which increase total spending.

The in vitro conservation of plant genetic resources involves the storage of cell lines and plant tissues such as meristems, somatic embryoids, zygotic embryos, microshoots, and pollen under sterile conditions. Plant material may be stored for several months to several years in in vitro germplasm conservation programs, but this process requires highly skilled labor and advanced research facilities [16]. However, there is a need to fine-tune the protocol for in vitro conservation for mid and long-term conservation [17]. There is also a need for a genotype-independent somatic embryogenesis protocol to conserve the somatic embryoids. The plant material (calli, somatic embryoids, and meristem) is pest-free and safe to be moved under phytosanitary rules [18]. In vitro germplasm conservation through cryopreservation may help prevent genetic erosion and conserve palm genetic resources. Moreover, it could help conserve plant germplasm in a safe environment and store it for several years. However, the procedures involved in in vitro conservation methods are expensive, require specialized infrastructure and expertise, and a continuous supply of expendable materials.

Ex situ germplasm conservation faces several limitations, including the progressive loss of genetic diversity and the costs and labor required to maintain germplasm collections. Therefore, ex situ germplasm conservation may be augmented by in vitro conservation.

## 5. Seed Bank Storage

Seeds can be classified into three distinct categories (orthodox, intermediate, and recalcitrant) based on their response to storage conditions. The species respond differently to seed tissue desiccation, depending on seed type (orthodox, intermediate, or recalcitrant), and exhibit different glass-forming states. Oil palm is considered to fall within the intermediate category, exhibiting characteristics that place it between orthodox and recalcitrant types in terms of storage tolerance [19]. The species respond differently to seed tissue desiccation, depending on seed type (orthodox, intermediate, or recalcitrant), and exhibit different glass-forming states. Specifically, oil palm intact seeds lose viability more rapidly at moisture contents of 6–7% when stored at 0 °C and −20 °C, reducing storage time [20]. Moreover, a few accessions of oil palm lost their seed viability when they were desiccated to 4–5% [19].

Seed banks can be established for short- to mid-term conservation, depending on the seed desiccation level (5–8%) and storage temperature below 4 °C [21]. The seeds can be stored for at least 3 months under controlled conditions at 22–28 °C and 11% moisture content [20]. Moreover, seed (10–12%) and embryo (19–21%) moisture contents can be stored for 12 months at 15 °C under hermetic storage conditions [19]. However, under ambient storage conditions, the seeds can only be stored for about two months [20]. Seed germination progressively decreased from 0–6 months when stored under ambient storage conditions, and seedling establishment into a young plant is characterized by a slow growth rate due to prolonged seed dormancy [22,23].

Seed may undergo rapid biochemical (lipid peroxidation, protein degradation, and enzyme activation (lipases, amylases, and proteases), reactive oxygen species), physiological changes (respiration, desiccation), and microbial contamination during storage, resulting in a decline in viability over time [20,24,25]. The seeds are rich in oil, and high oil content can lead to oxidation in the endosperm, which deteriorates cellular structures, damages the embryo, and loses viability when stored under ambient conditions [20]. Seed storage also poses several challenges, including the need to regenerate seed regularly due to loss of viability and poor shelf life. There are also changes in allele frequency due to seed contamination (mechanical mixtures and outbreeding) resulting from repeated cultivation of germplasm in a short-term germplasm conservation program.

While seed banks are suitable for short to medium term conservation, the long term conservation of seed or kernel is difficult and challenging due to poor desiccation tolerance and low temperature, as the species’ seed falls in the intermediate category. In vitro conservation provides a more effective means of germplasm. Cryopreservation is particularly suitable for recalcitrant or intermediate desiccation-intolerant species that lose viability in seed banks. However, in vitro protocols must be optimized through extensive experimentation, and the regeneration of explants or propagules after cryopreservation must also be optimized.

## 6. In Vitro Conservation and Plant Tissue Culture

In vitro conservation techniques are based on tissue culture protocols that enable the regeneration of pathogen-free plants from meristems or polyembryoids under aseptic conditions. These tissue culture protocols are aseptic methods for clonal propagation, allowing the regeneration of virtually any genotype from totipotent cells, tissues, or organs. In this process, cultured explants form an undifferentiated mass of cells known as ‘calli.’ The regeneration process includes direct and indirect somatic embryogenesis [26,27]. Direct embryogenesis involves the formation of somatic embryos in the explant, bypassing the callus phase, provided the explant and growth media are appropriate [26]. Indirect somatic embryogenesis, in which somatic embryoids occur in calli, where embryogenic calli are induced using specific concentrations of nutrient media and plant growth regulators [28]. Optimized culture media and growth conditions facilitate the development of heart-shaped structures known as ‘somatic embryos. These somatic embryos mature, germinate, and produce viable shoots, thereby completing the regeneration process. A key prerequisite for in vitro conservation is the optimization of a highly efficient, regenerable somatic embryogenesis protocol. Different explants and media protocols were optimized to obtain regeneration through somatic embryogenesis in the optimized oil palm tissue culture [28]. Somatic embryogenesis is influenced by genotype rather than by the concentration of the media, plant growth regulators, or types of explants, which is a significant impediment to germplasm conservation, highlighting the need to develop a genotype-independent regeneration protocol to ensure the successful conservation of diverse germplasm sets [29].

**Figure 2:**
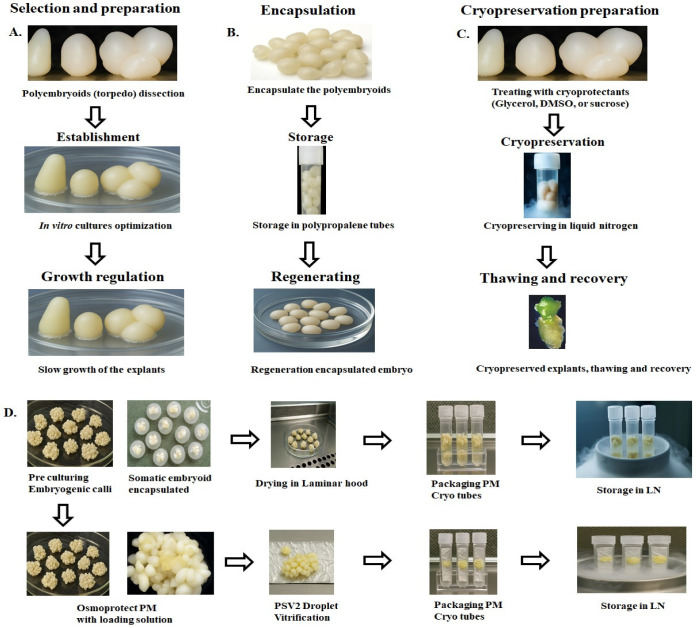
Overall view of in vitro conservation of germplasm (**A**). Somatic polyembryoids in slow-growth media (**B**). Medium term storage of zygotic and somatic embryoids under low temperature (**C**). Long term storage of zygotic and somatic embryoids (**D**). Various steps of vitrification and cryogenic conservation of plant material (PM) in liquid nitrogen (LN).

Several types of explants, such as zygotic embryos, leaves, and inflorescences, have been used in tissue culture, with varying success rates. Zygotic embryos, for example, are known for their high callus-induction rate. However, zygotic embryos may not guarantee 100% genetic fidelity to their maternal plants. Recently, a protocol for callus induction and somatic embryogenesis from various explants has been published [28]. In this study, explants were obtained from the heart of the palm, and different leaf segments (L1–L8) were inoculated onto growth media. Leaf segments (L2–L8) exhibited a 10% callus formation rate, whereas somatic embryoids incubated in the RITA bioreactor with suitable plantlet regeneration growth media achieved a success rate of 5% [28]. Embryogenic calli containing polyembryoids are subjected to in vitro conservation through slow growth or cryogenic preservation (Figure 2).

A variety of calli, including compact granular and compact green, can be obtained from various concentrations of growth media and plant growth regulators. Calli are proliferated in suspension or bioreactors, such as temporary immersion systems. Somatic cells are generally cultured in liquid Murashige & Skoog (MS) media with 83 mM sucrose and 0.54 µM 2,4-D. These calli can be compact nodular, friable, or friable nodular. Studies indicate that compact nodular calli exhibit high regeneration efficiency and can be utilized in slow-growth media or for the cryopreservation of polyembryoid clumps. These polyembryonic aggregates, along with developing embryoids, should be greater than 500 µm in diameter and should be selected from the cell suspension [17]. Moreover, embryogenic calli with torpedo structures or haustoria are essential for the regression of polyembryoids after storage. In conclusion, embryogenic compact nodular calli are the most suitable material for in vitro conservation through slower growth or cryopreservation. These calli are highly regenerable and maintain genetic fidelity after preservation.

Methods of in vitro conservation can be practiced for mid-term storage, while cryopreservation is done for long-term storage (Figure 2). Typically, in vitro meristematic tissues or somatic embryoids are inoculated into slow-growth media or cryopreserved to sustain viability for medium- to long-term preservation. Medium-term storage is achieved by inducing slow growth through the retardation of minimal growth media, using growth retardants or low temperatures [30]. This enables prolonging subculturing intervals from 6 to 24 months. Subsequent increases in subculturing cycles reduce the resources and labor required for germplasm maintenance. “Cryopreservation” is a long-term storage method that requires samples to be kept under cryogenic conditions in liquid nitrogen to maintain viability and integrity for extended periods [30]. The procedure is expensive, complex, and time-consuming, requiring significant labor, resources, and expertise.

### 6.1. Factors Affecting In Vitro Conservation

Medium-term germplasm storage can be readily achieved using standard procedures used in plant tissue culture laboratories. However, the optimal procedure for propagule regeneration may be required before germplasm is subjected to in vitro conservation. A key consideration in medium- or long-term conservation methods is to slow or retard the growth of the cultures, while maintaining good viability during and after storage to ensure the successful recovery of conserved plant material. The following factors are optimized in protocols for in vitro germplasm conservation.

#### 6.1.1. Growth Conditions

The factors considered include the photoperiod (or dark period), temperature, gaseous exchange, and media composition [31,32]. These factors were optimized individually or in combination for particular species. Temperature is critical during short- or medium-term storage, as it helps slow explant growth [33]. Low temperature is the most common medium-term strategy for conserving germplasm; however, tropical plant species, such as oil palms, are sensitive to low temperatures and require storage at higher temperatures. The storage temperature for cool-season crops ranges from 0 to 5 °C, while for tropical species, the optimum storage temperature is 10 to 20 °C, depending on the species [33].

#### 6.1.2. Osmotics and Encapsulation

Specialized growth media are tailored for the in vitro preservation of shoots, promoting slow explant growth. This gradual growth helps reduce the need for frequent subculturing by limiting cell division and growth, thereby maintaining genetic stability [13]. As a result, cultures can be sustained for several years with minimal subculturing, depending on the plant species [17]. In vitro conservation of oil palm germplasm was performed at 20–25 °C using half-strength MS media supplemented with 3% mannitol. This method effectively inhibited the growth of aerial parts more than other osmotic agents, such as sorbitol and sucrose, achieving a 100% survival rate after 1 year of in vitro conservation [34]. In comparison to the various osmotica (mannitol, sorbitol, and sucrose), the selection process was complicated by varying responses of palm cultivars to different concentrations of osmotica, making it challenging to identify a single optimal concentration effective across all cultivars [34]. However, the study revealed that 3% mannitol provided better survival rates and better control over the growth of the aerial parts in oil palm [34]. Oil palm polyembryoids at the developmental stage of torpedo-shaped structures and haustoria were encapsulated with 3% sodium alginate by dipping in calcium-free MS media [35]. The 6–7 mm beads encapsulating the polyembryoids were polymerized in 100 mL of 75 mM CaCl_2_, which polymerizes the sodium alginate. The translucent coat was visually observed and turned opaque upon completion of the polymerization process [35]. These encapsulated synthetic oil palm seeds were stored at temperatures ranging between 5 and 25 °C for up to 70 days. However, after just two months of storage, synthetic seeds lost their viability at high temperatures, especially at 25 °C. The best storage temperature was 5 °C, with the synthetic seeds retaining 67% viability, compared to 0% at 25 °C and 20% at 10 °C [35].

#### 6.1.3. Nutrient Media

Modifications in growth media, such as reducing the concentration of minerals, sucrose, and types of growth regulators, along with the use of chemicals to lower osmotic potentials (e.g., mannitol or polyethylene glycol), have also been beneficial for inducing slow growth [30].

Growth retardants act as signals and precursors of growth and development. They typically bind to receptors in the cell, inducing various responses by modifying gene expression. These retardants activate novel gene expression and block normal growth and development. Different growth retardants, such as the gibberellin inhibitor paclobutrazol (PBZ 0–10.34 μM) and the tranexamic acid ethyl ester (TNE 0–13.7 μM), have been applied to reduce growth during in vitro conservation. A study on non-palm species showed that concentrations of 3.44 μM (PBZ) and 4.56 μM (TNE) successfully retarded the growth of the explant aerial parts without affecting the species’ survivability (96%) even after 180 days of culture [36].

In date palm (*Phoenix dactylifera* L.), MS media supplemented with 2 mg/L abscisic acid and an osmotic concentration of 90 mg/L sucrose successfully increased the survivability of germplasm without the need for subculturing for up to 10 months at 18 °C and a light intensity of 10 μ mol m^2^ s^−1^ [37].

The size and volume of culture vessels were also crucial for storing explants. Activated charcoal was used to control the secretion of substances from the explants. The culture media were modified to include vitamins and antibiotics to prevent bacterial growth. Dormancy and bacterial growth were minimized by carefully selecting protocols and optimizing the subculturing process for oil palm explants.

## 7. Cryopreservation

Tissues or cells can be cryopreserved indefinitely in liquid nitrogen, allowing long-term storage without loss of viability or genetic stability of the preserved germplasm. Cryopreservation inhibits all metabolic activities and cell division, thereby maintaining the germplasm indefinitely [38]. However, protocols may be fine-tuned to have minimal effects on oil palm genetic or phenotypic characteristics [39]. Cryopreservation protocols for oil palm have been established to prevent explant damage while preserving their regeneration capacity after rewarming and rehydration [31,40] (Figure 2). The effects of various factors, including the pre-culture and loading solution, on explant survival are documented in Table 1.

Generally, tissues with high water content are not suitable for cryopreservation. Additionally, dehydration must be rapid and uniform to avoid intracellular ice formation. Therefore, small-sized explants are recommended for more successful cryopreservation of genetic material. Meristematic and embryonic tissues, shoot tips, somatic embryoids, pollen, and zygotic embryos are commonly considered for in vitro cryopreservation [38,41]. Tissues exhibit varying responses to cryopreservation, and cultivars within species also respond differentially, necessitating the optimization of genotype-independent protocols.

Both penetrating and non-penetrating cryoprotectants are used to prevent injuries during dehydration. Various vitrification protocols, such as PVS2 and PVS3, have been developed for the cryopreservation of somatic embryoids and apices in tropical and temperate species [17]. PVS2 has been successfully applied in cryopreserving apical meristem tissues, calluses, cells, and somatic embryoids in clonally propagated species.

Encapsulation and dehydration techniques, which involve embedding plant material in beads, protect against dehydration and freezing damage. This technique has been successfully applied in clonally propagated species [42]. Somatic embryoids and zygotic embryos are often used for preservation, with factors such as sucrose concentration, dehydration period, and freezing damage carefully considered during cryopreservation [39].

**Table 1:** Effect of various factors, pre-culturing, and loading solutions on the survival of the explant.

Explant	Survival Rate	Preculturing	Loading Solution	Factors	Reference
Somatic embryoids (torpedo stage)	73%	Preculturing sucrose (0.3–1 M)	3% (*w*/*v*) sodium alginate and 100 mM CaCl_2_	Encapsulated 3% sodium alginate and 100 mM CaCl_2_ Drying: 9 h, Moisture content 23%	[35]
Somatic embryoids (torpedo stage)	68%	Sucrose 0.5 M for 12 h	[10% (*w*/*v*) dimethyl sulphoxide (DMSO) plus 0.7 M sucrose] 30 min	Drying for 5 min	
Somatic embryoids (clumps)	33%	Sucrose 0.75 M		28 surviving clones, 3 lost due to contamination. Regenerated after 20 years	[43]
Somatic embryoids	45%	0.5 M 12 h	Several loading solutions	L5 was the best loading solution while PVS2 gave the highest survival%. Exposure time was 5 min for the PVS2 before LN storage	[42]
Zygotic embryos	80%	Moisture 0.16 g DW^−1^	-	Zygotic embryos were put in 1.5 mL cryotubes and then plunged into liquid nitrogen.	[41,44]
Zygotic embryo	96%	Rehydration + 0.12 g H_2_O/g DW over silica gel	sterile 2 mL polypropylene cryotubes	Increased survival of the embryo with rehydration and desiccation treatment	[45]
Zygotic embryo	71%	No rehydration	sterile 2 mL polypropylene cryotubes	Low survival of control kernels as no rehydration treatment was applied	[45]
Zygotic embryo	85%	Long dehydration and desiccation to 0.30 g H_2_O/g	sterile 2 mL polypropylene cryotubes	Very high development rate after cryopreservation (65%)	[45]
Somatic embryoids	50–100%	16 h dessication under silixa gel	Liquid nitrogen	Pre-culture for 7 days on MS media with 0.75 M sucrose and desiccation on silica gel	[46]

### 7.1. Pre-Culturing of Somatic Embryoids

Somatic embryoid clumps, zygotic embryos, pollen, seeds, and kernels have all been explored for cryopreservation. However, the most successful methods have involved somatic or zygotic embryoids, which exhibit higher viability and survival rates following cryopreservation and thawing [38,41]. The initial protocol for the cryopreservation of the somatic embryoids had survival rates of 31–55% while the recovery rate was 10–20% [47]. This was improved through pre-culture techniques involving desiccation in a concentrated sucrose solution, reaching survival up to 80% [47]. The protocol for cryopreservation of somatic embryoids at the torpedo stage was established by Engelmann [31]. These somatic embryoids were produced through two-month cultures with 0.3 M sucrose as the carbon source, which proved more effective than mannitol and sorbitol. Immature zygotic embryos and their endosperm were dehydrated for 3 days, placed in 2 mL cryopreservation tubes, and stored in liquid nitrogen for 1–60 days. The samples were thawed in a water bath at 40 °C for 30 min. Zygotic embryos were dissected and cultured on Y3 media supplemented with 87.6 mM sucrose and 2.67 μM phytagel. They exhibited over 93% viability and 84% germination, whereas the somatic embryoids showed only 68% viability after cryopreservation [39].

Somatic embryoids from 28 clones, regenerated after 20 years of cryopreservation, showed an overall average survivability of 33% [43]. However, three clones were lost due to contamination and poor in vitro growth. Pollen was first cryopreserved in 1998. Pollen viability was assessed using the fluorescein diacetate (FDA) test and in vitro germination. The in vitro germination test media included 73.03 mM sucrose, 1.615 mM boric acid, and 10% polyethylene glycol. Pollen viability and in vitro germination rates were 62% and 52% for both procedures [48], with a moisture content of 23.3% during storage. They were removed in 2008, thawed, and warmed at 38 °C for 5 min, after which their pollen viability was evaluated. This was statistically insignificant compared to the original, while in vitro pollen germination was 49% [48].

Meristematic tissues are also preferred explants for in vitro conservation of germplasm in some species. Micromeristematic tissues, excised under aseptic conditions, are generally considered free from viruses and bacteria. Moreover, these tissues produce true-to-type plants and are less prone to mutations than callus or cell cultures [49]. However, meristematic tissues in oil palm may not be suitable due to their limited availability, and their excision may lead to the loss of the entire plant. Clonal propagation of oil palm is significantly more challenging than that of other palm species, such as date palm, because oil palms do not produce offshoots. Historically, oil palms were cultivated from seeds before the optimization of somatic embryogenesis protocols.

### 7.2. Encapsulation-Dehydration

The protocol regarding encapsulation- dehydration has been described in detail in the book chapter [50]. The method is based on techniques for encapsulation with sodium or calcium alginate for synthetic seeds. Encapsulated explants, such as somatic embryoids, are protected from extreme conditions of high osmotic stress induced by sucrose during preculture and desiccation [50]. These treatments are applied before storage in liquid nitrogen (LN). The desiccation of the explants replaces the explant with loading solutes to prevent freezing during rapid exposure to LN, thereby avoiding lethal intracellular ice crystallization. Therefore, explant remains viable after rewarming, resulting in high survival, and direct regrowth cryopreservation [50].Embryos of oil palm were encapsulated in alginate beads to protect them from direct exposure to vitrification solutions, thus safeguarding against the harmful effects of rapid dehydration. Initially, somatic embryoids were pre-cultured in a sucrose solution ranging from 0.3 to 1 M for 7 days. They were subsequently encapsulated in 3% sodium alginate together with 100 mM CaCl2, followed by a drying period of 9 h that lowered their moisture content to 23%. The dehydrated encapsulated embryoids were then placed in cryotubes, stored in liquid nitrogen for 1 h, thawed, and cultured on regeneration media. The cryopreserved and thawed polyembryoids showed a survival rate of 73% [35]. Moreover, somatic embryoids were dried to a moisture content of 22% before undergoing cryopreservation for a period of 20 years [43].

### 7.3. Vitrification

Vitrification is the physical process by which a concentrated solution transforms into a metastable glass under very low temperatures without crystallization [51]. The theory of vitrification aims to convert tissues into a vitrified state without forming intra- or extracellular ice. Ice crystals formed due to improper handling can damage the propagule, leading to loss of tissue viability. The vitrification process requires careful selection and concentration of vitrification salts to avoid injury or salt toxicity during desiccation. Vitrification-based cryopreservation is the most common method, and several protocol modifications exist, including encapsulated vitrification, droplet vitrification, V-cryoplate, and D-cryoplate. Several vitrification solutions have been proposed, which are presented in Table 2. Vitrification has been regarded as a powerful tool in numerous studies conducted on oil palm cryopreservation [52]. It has been reviewed that vitrification is the most reliable method for preserving oil palm germplasm, particularly through somatic embryos, zygotic embryoids, seeds, pollen, and embryogenic suspensions [47]. The duration of exposure to various vitrification solutions, such as PVS2, is a critical factor, as different studies have shown that polyembryoid survivability declines with increasing exposure time. A 5 min exposure induced vitrification in samples [42]. In another study, PVS2 was recommended for 10 min for oil palm. In another study, somatic embryoids were dehydrated in a highly concentrated PSV2 solution for 30 min at 0 °C, with a 66% survival rate after liquid nitrogen cryopreservation [52]. The list of preservation media is documented in Table 2. Among the loading solutions, L5 treated for 30 min was considered closest to the control due to its minimal detrimental effects on somatic embryoid viability [42].

**Table 2:** List of loading and vitrification solutions used in oil palm.

Code	Recipe	References
Loading solution		
L1	[2.0 M glycerol + 0.4 M sucrose]	[53]
L2	[1.5 M glycerol + 0.4 M sucrose + 5% (*w*/*v*) (DMSO *)]	[54]
L3	[0.5 M glycerol + 0.3 M sucrose + 10% (*w*/*v*) DMSO]	[55]
L4	[30% (*w*/*v*) glycerol + 15% (*w*/*v*) ethylene glycol (EG) + 15% DMSO + 0.4 M sucrose]	[56]
L5	[10% (*w*/*v*) DMSO + 0.7 M sucrose]	
Plant Vitrification Solutions		
PVS	[22% (*w*/*v*) glycerol + 15% (*w*/*v*) EG + 15% (*w*/*v*) propylene glycol + 7% (*w*/*v*) DMSO + 0.5 M sorbitol]	[57]
PVS2	[30% (*w*/*v*) glycerol + 15% (*w*/*v*) EG + 15% (*w*/*v*) DMSO + 0.4 M sucrose	[56]
PVS3	[50% (*w*/*v*) glycerol + 50% sucrose]	[58]
L-solution	[22% (*w*/*v*) glycerol + 30% (*w*/*v*) EG + 7% (*w*/*v*) DMSO + 15% (*w*/*v*) sucrose + 10 m CaCl_2_]	[59]
Towill solution	EG + 1 M DMSO + 10% PEG	[60]
Watanabe solution	[44.5% (*w*/*v*) DMSO + 18.7% (*w*/*v*) sorbitol for 10 min]	[61]

* Dimethyl sulfoxide (DMSO).

### 7.4. Droplet Vitrification

This is a modified vitrification technique that utilizes small droplets of cryoprotectants, with the samples placed on aluminum foils. Rapid cooling of the samples is achieved by directly exposing them to liquid nitrogen. Cryoprotectants help maintain the samples in the form of droplets. Microdroplets freeze spontaneously to a temperature below the glass transition phase, forming glass without ice crystal formation. This method has been used for the cryopreservation of polyembryoids and is known as V-cryoplates in the context of droplet vitrification. The primary advantage of this technique is the rapid cooling of the sample, which allows direct transition to the glassy state. Additionally, microdroplet formation enables the cryopreservation of small-sized samples, such as cells, somatic embryoids, and torpedoes, and is more effective than other vitrification methods. Pre-cultured polyembryoids were treated with a loading solution of 1.5 M glycerol, 0.4 M sucrose, and 5% (*w*/*v*) DMSO for 30 min under continuous light at 60 μ mol m^−2^ s^−1^ photosynthetically active photon flux density (PPFD) at 26 °C [42]. Polyembryoids precultured on 0.5 M sucrose and treated with the loading solution were then subjected to pre-chilled 100 µL of PVS2 vitrification solution, with varying drying durations. A 10 min drying period reduced the moisture content to 47%, and the survival rate was approximately 68% after exposure to liquid nitrogen [49].

## 8. Genetic Modification Leading to Somaclonal Variations

The genetic stability of conserved cultivars is crucial, as species are prone to mutations during callus formation and during long-term cryopreservation [58]. The genetic stability of regenerated germplasm can be determined using cytological, molecular, and morphological markers [62]. Marker-assisted germplasm screening may also be conducted to confirm genetic stability and estimate changes in gene frequency [35,63]. The genetic similarities between mother shoots and their regenerated plantlets ranged from 83% (R5) to 94% (R4) [64].

The activation of abiotic stress or cryotolerance genes has been reported in oil palm [39]. These genes are associated with activating the reactive oxygen species scavenger enzyme system, specifically in somatic embryoids [39]. Markers sensitive to methylation can be used to amplify polymorphisms, which, in turn, can help determine epigenetic changes during somatic embryogenesis and cryopreservation [65]. Tissue culture techniques may also induce epigenetic changes within somatic embryoids; however, these changes are less intense than those observed in somatic embryoids regenerated after cryopreservation in tropical plant species, such as cocoa. These epigenetic changes are reversible as plants progress from seedlings to mature plants [66]. Generally, genomic modifications ranging from 0% to 10% have been reported in various crop species, whereas methylation levels of 0.5% to 7% have been detected through molecular markers in different crops [67]. Low genetic modification was induced in other palm species during the cryopreservation of 12 date palm cultivars. Genetic differentiation was detected using 12 start codon target polymorphism (SCoT) markers, and most alleles were found to be monomorphic within the regenerated cultivars [65]. Additional research may be necessary, utilizing SNP markers or whole-genome sequencing to identify potential genomic sites susceptible to modifications or mutations, which could enhance our understanding of the genetic stability of stored material [68].

## 9. Problems with Cryopreservation and the Way Forward

The major problem with cryopreservation is the genotype specificity for somatic embryogenesis and its variable responses to cryopreservation. Each genotype comprises a specific set of alleles in the genome that help it respond to the in vitro conditions for somatic embryogenesis. Several genes related to somatic embryogenesis, specifically *15 WUSCHEL-related homeobox* (*WOX*) genes and *BROOM*, have been identified in the genome of oil palm [69]. Other genes related to somatic embryogenesis include *EgIAA9*, *EgHOX1*, *EgLSD*, and *EgER6*, which are activated by indole acetic acid and other growth regulators [70,71,72]. There is a need to understand the upregulation of genes related to somatic embryogenesis, and fine-tuning their expression may help induce somatic embryogenesis in recalcitrant types. Moreover, epigenetic factors related to callus induction and somatic embryogenesis may also be considered, as it was identified that procambial cells of leaf sheath undergo hypomethylation (5%) triggered by indole acetic acid. In contrast, non-responsive cells (Parenchymatous sheath cells) show contrasting behavior, i.e., hypermethylation [73]. Methylation-sensitive DNA markers may be used to differentiate the responsive and non-responsive cellular genotypes. There is also the variable response of genotypes to cryopreservation, with few genotypes successfully preserved through this process, while others exhibit a recalcitrant response. This differential response may be due to the variability among germplasm accessions in their tolerance to adverse conditions, such as salt toxicity, chilling, and abiotic stress. Generally, stresses induce the leakage of the membrane due to reactive oxygen species (ROS), which activate ROS scavenging enzymes such as superoxide dismutase (SOD) and peroxidase (POD) [74]. The mechanism of abiotic stress tolerance may be fully understood, and strategies may be formulated. For instance, a study has shown that the expression of several cold-tolerant genes, including CBF1 and CBF2, is related to osmolytes such as proline and sucrose, while cold injury is associated with the accumulation of proline [75]. Moreover, secondary metabolites such as flavonoids, plant hormones, and vitexin, isovitexin, and apigenin 6-C-glucoside were accumulated in response to chilling stress and contributed to their antioxidant activities under stress conditions [74]. The synthesis of L-glutamic acid and sarcosine in resistant accessions under chilling stress aids in protein synthesis and supports cell recovery and repair mechanisms. The *EgCBF3* gene has been reported to be activated in response to several abiotic stresses through abscisic acid signaling [76]. Thus, inducing progressive treatment of stress or allowing polyembryoids to harden before cryopreservation, or exposing them to osmolytes and enhancing the media composition with antioxidants and amino acids, may help better acclimate them during cryopreservation.

## 10. Future Outlook and Conclusions

Advances in cryopreservation have revolutionized the conservation of germplasm. Newly optimized techniques, such as vitrification and encapsulation-dehydration, have proven highly effective for conserving somatic and zygotic embryos. Optimizing cryoprotectants, such as DMSO and sucrose solutions, has significantly reduced the toxicity and osmotic stress experienced by embryos, thereby increasing the overall success of long-term storage. The success of cryopreservation may play a significant role in conserving genetic diversity, particularly in oil palm. All kinds of genetic variability, such as obsolete cultivars or wild species, and hybrids may be conserved for longer times. The cryopreservation could help oil palm breeding programs by conserving mapping populations or core germplasm sets containing valuable traits over extended periods. This may reduce the costs associated with preserving intensive field gene banks. It is also a convenient method for transferring and exchanging plant materials across various geographical regions, thereby contributing to the conservation of global genetic resources. This may provide a stable foundation for the long-term genetic improvement of oil palm.

This technology will not only help conserve genetic diversity but also facilitate molecular research. For instance, the vitrified embryo has been a viable alternative to the fresh zygotic embryo for CRISPR/Cas9 microinjection, with a survival rate of 63% compared to 49% and an almost comparable mutation rate.

The real challenge for future research may be to explore the non-toxic alternative DMSO, which could provide a significantly higher protection against cryoinjury and potentially lead to better survival rates. Encapsulation-dehydration methods combined with cryopreservation can protect against dehydration and ice formation, which is crucial for maintaining tissue viability. Furthermore, the genetic stability of the conserved plant material must be maintained and not compromised during storage. The use of genetic modification technology may be exploited to modify genes or disrupt the mechanisms that induce injuries during cryopreservation. The bioreactor systems can also be utilized for the mass production of somatic embryoids, which can later be stored, thereby enhancing our efforts in large-scale production and cryopreservation. This could be a game-changer for the large-scale conservation efforts.

The major shortcoming of cryopreservation of somatic embryoids is the genotype-specificity of somatic embryogenesis, which may hinder large-scale production in targeted accessions. Research has shown that the success of cryopreservation has been applied to a limited number of genotypes within species, and there is significant variability among these genotypes in terms of their totipotency potential. Moreover, the cultivars also exhibit variable responses to dehydration and cryoprotectant treatment. This genotype dependency complicates the development of universal protocols for oil palm cryopreservation. Understanding the genetic and physiological factors that affect genotypic responses to cryopreservation is required, as it may enable the development of standardized, genotype-independent protocols. There is also a need to understand the mechanisms behind cryoinjuries and how to mitigate them effectively, a topic that remains underexplored. Therefore, research is needed to know how to prevent damage caused by ice formation and osmotic stress during the cryopreservation process.

In conclusion, in vitro conservation is a potential technique for preserving genetic diversity within a plant species. This technique has been exploited to preserve polyembryoids for medium- to long-term storage in oil palm. Polyembryoids were subjected to mid-term conservation at low temperatures, with a slow growth rate induced by different osmotica. In certain instances, they were encapsulated in sodium alginate to enhance low-temperature tolerance before storage. In long-term conservation, the polyembryoids were desiccated and precultured on various osmotica before being subjected to the loading solution. Moreover, polyembryoids were subjected to vitrification, including droplet vitrification, before cryopreservation. The cryopreserved embryoids were thawed and cultured in regeneration media, yielding variable results in terms of survival and growth. The somatic embryogenesis process is strongly genotype-dependent, and the composition of the growth media and concentration of plant growth regulators also influence its success. Thereby, the applicability of this technique is limited to a restricted number of genotypes, which can reduce its usefulness. The genetic stability of the genotypes can be determined through advanced molecular techniques, such as genome sequencing, to identify potential sites of mutations and genome modifications that may occur during storage. This enables the detection of any genetic changes that may have happened, ensuring that the conserved plant material remains genetically stable and accurately represents its type.

## Data Availability

No new data were created or analyzed in this study. Data sharing is not applicable to this article.
